# Magnetic resonance-guided focused ultrasound for uterine fibroids

**DOI:** 10.1016/j.clinsp.2025.100603

**Published:** 2025-03-04

**Authors:** Pedro Felipe Magalhães Peregrino, Marcos de Lorenzo Messina, José Maria Soares Júnior, Edmund Chada Baracat

**Affiliations:** Gynecology Discipline, Department of Obstetrics and Gynecology, Hospital das Clínicas da Faculdade de Medicina da Universidade de São Paulo (HCFMUSP), São Paulo, SP, Brazil

**Keywords:** Myoma, Leiomyoma, Fibroid, High-intensity focused ultrasound ablation, HIFU, MRgFUS

## Abstract

**Introduction:**

The uterine leiomyoma is a pelvic solid tumor more frequent in women. The Magnetic Resonance-Guided Focused Ultrasound (MRgFUS) is a technique aimed at diminishing hypermenorrhagia and dysmenorrhea in symptomatic women.

**Objective:**

To show the effects subsequent to MRgFUS application on symptoms, quality of life, and myoma volume in symptomatic women.

**Methods:**

Fifty-two patients with symptomatic uterine myomas underwent MRgFUS followed by clinical evaluation, pelvis MRI, and administration of the UFS-QoL questionnaire at 6 months and 12 months to detect symptom improvements and decrease in myoma volume.

**Results:**

The mean myoma volume decreased 24 % after 6 months and 42 % after 12 months. The mean score on the UFS-QoL questionnaire increased 54 % after 6 months and 81 % after 12 months. There were no serious complications. Four patients had first-degree burns. No infection cases were reported. Two patients progressed to a myomectomy by surgical hysteroscopy and one of them underwent total hysterectomy 11 months after the MRgFUS treatment.

**Conclusion:**

This case series suggests that MRgFUS therapy is viable and effective in reducing symptoms and improving the life quality of symptomatic patients.

## Highlights

• Fibroids are tumors that can cause important symptoms that reduce women's quality of life.
• The gold standard treatment for symptomatic fibroids is hysterectomy.
• Myomectomy is the treatment of choice for women with reproductive desire.
• Magnetic resonance-guided focused ultrasound for uterine fibroids is a minimally invasive treatment alternative for symptomatic women.
• Magnetic resonance-guided focused ultrasound for uterine fibroids proves to be a good alternative to improve the symptoms and quality of life of symptomatic women.

## Introduction

A leiomyoma is a benign neoplasm that originates from smooth muscle tissue and, according to its location, is classified as subserosal, intramural, or submucosal. These types of tumors manifest in diverse ways. The main symptoms are hypermenorrhagia, dysmenorrhea, dyspareunia, compression of adjacent organs (bladder and intestines), increased abdominal volume, infertility, and recurrent miscarriage.^1^, ^2^, ^3^ Hypermenorrhagia may cause anemia, which, along with dysmenorrhea and dyspareunia, impairs quality of life. The increase in abdominal volume and the compression of adjacent organs may change intestinal and urinary habits, besides causing women aesthetic discomfort.^3^

The classical treatment of leiomyoma is myomectomy or hysterectomy. However, these techniques may be very costly because of surgical time, increased bleeding risk, longer hospital stays, and possible postoperative complications. Therefore, researchers are developing new techniques, such as the Magnetic Resonance-Guided Focused Ultrasound (MRgFUS).^4^, ^5^, ^6^

The MRgFUS is a minimally invasive therapeutic option capable of producing necrosis through thermal coagulation of myometrial nodules at a precise point in the uterus.^4^ Its main objective is to reduce fibroid volume and mitigate the complaints about symptoms. However, is not an established procedure as an alternative therapy in many centers. Nevertheless, initial studies have shown a variety of cases with considerable improvement in symptoms. Even with partial results, the MRgFUS has demonstrated it is a minimally invasive method that is efficient in the treatment of myomas.^4^, ^5^, ^6^ This article reports a series of cases of patients with symptomatic uterine myomas who underwent treatment with MRgFUS for assessment of this technique.

## Methods

From January 2018 to December 2020, sixty-three patients with symptomatic uterine myomas were selected from among the patients at the Setor de Mioma Uterino da Divisão de Clínica Ginecológica do Hospital das Clínicas da Faculdade de Medicina da Universidade de São Paulo (HCFMUSP).

### Patients

The inclusion criteria for the patients were as follows: minimal age of 18-years and maximum age of 49-years; premenopausal women with no desire for children; the presence of symptomatic uterine myoma (abnormal uterine bleeding, pelvic pain, and increased abdominal volume); the presence of 2.5 to 10 cm myomas; absence of treatment with a GnRH analog, estrogen, or progesterone in the previous 3-months; and a free and informed consent statement.

### Clinical evaluation

The initial evaluation consisted of the following: questions about demographics, parity, and presence of comorbidities; a physical examination; a pelvic MRI; and the UFS-QoL questionnaire.^7^ After the treatment, another clinical evaluation was performed at 6 months, and still another at 12-months.

### The UFS-QoL questionnaire (Quality of life and symptoms of uterine myoma)

The UFS-QoL questionnaire assesses the symptoms of uterine myomas and the patient's quality of life. There are eight questions about the type and severity of symptoms. The answers are given on a 5-point response scale ranging from “no pain” (1) to “a lot” (5) and then converted into a Symptom Severity Score (SSS). Next, there are 29 questions about how the disease impacts different aspects of the patient's health-related quality of life. The responses checked on the 5-point scale from “none of the time” (1) to “all of the time” (5) are again converted into a score. Additionally, the patient answers questions about her concerns, activities, energy/mood, control, self-awareness, and sexual function.^7^

### Pelvic magnetic resonance imaging

The pelvic MRI for assessing uterine volume and characteristics of the myomas was conducted at the Departamento de Radiologia do Instituto do Câncer do Estado de São Paulo da FMUSP (ICESP) or at the Instituto de Radiologia do Hospital das Clínicas da Faculdade de Medicina da USP.

### Magnetic resonance-guided focused ultrasound

The MRgFUS procedure was carried out with the ExAblate® device at the Instituto do Câncer do Estado de São Paulo da FMUSP, following the techniques described below:

1. Indwelling bladder catheterization was carried out with a Foley catheter.
2. Heart rate and pO2 were monitored throughout the procedure using standard monitoring MRI-compatible devices.
3. The hair located along the path of the ultrasound beam was shaved and the skin was cleaned with an alcohol antiseptic in case there was an oil-based product on the skin.
4. The patient's skin in the treatment area was carefully examined for scars.
5. An MRI scan was performed with T2- or T1-weighted sequences in all three orientations to locate and evaluate the treatment area.
6. The anatomy in the treatment area was evaluated to identify any details that could prevent a safe treatment. The adjacent organs were evaluated to make sure bowel loops or a bladder region or important nerve bundles were not close to the treatment area or that no other obstruction stood in the way of the ultrasound beam.
7. The physician marked the target area in the MRI beam path. The drawing was larger than the target area itself to allow the physician to add (if necessary) additional sonication points during the treatment.
8. Using MRI, the physician outlined the treatment volume from one or more scan orientations.
9. A central point in the target area was sonicated with a low thermal dose, generating a subtherapeutic sonication in order to reconfirm target precision in the patient. The transducer positioning and the target positioning were adjusted as necessary.
10. As part of the treatment procedure, geometry verification in soft tissue was carried out to confirm the accuracy of the thermal location of the planned sonication point. Geometry verification was used to correct any residual geometric errors.
11. Before the performance of the treatment sonication, the patient received sedation and analgesia with Fentanyl and Midazolam.
12. Sonication's was performed at successive points (sonication duration between 5 and 60 ss). The effect of each sonication was measured with MRI using phase map images, reflecting temperature-dependent changes in resonant frequency. Sound power was adjusted during treatment to reach a maximum tissue temperature between 65 °C and 85 °C at the ablation target.
13. From the first to the last sonication, the total time was limited to 180 mins or to the patient's tolerance.
14. Immediately after treatment, a series of MRI scans (with and without contrasts) was performed. Scanning included T2-weighted sequences and T1-weighted contrast-enhanced sequences to measure the treatment effect.
15. After the procedure, the patient was moved to a recovery area where she was kept under observation until discharge.

### Statistical analysis

The statistical analyses were carried out with R (version 2.14.2). Changes in the SSS score, in the total UFS-QoL score, and in the initial uterine fibroid volume at 6 months and at 12 months of follow-up were assessed with the Friedman and the Wilcoxon tests. A p-value < 0.05 was considered statistically significant.

## Results

In this study, the primary outcomes underlining the effectiveness of the MRgFUS method were volume reduction of the uterus and uterine fibroids and improvement in symptoms, including the reintervention rate for persistent or recurrent symptoms. The volume of the uterus and that of the uterine fibroids were measured in longitudinal, anteroposterior, and transverse sections and were calculated according to the following formula: V¼ 0.5233 D1 × D2 × D3, where V stands for volume, D1 for longitudinal dimension, D2 for anteroposterior dimension, and D3 for transverse dimension. Improvement in menorrhagia was assessed with the UFS-QoL questionnaire; its score covers serious symptoms of vaginal bleeding and myoma-specific quality of life evaluation.^7^

Sixty-three premenopausal women were selected for this study. The mean age (± SD) of the patients was 38.52 ± 4.96 years (range: 27‒49 years). They had a total of 111 symptomatic uterine fibroids. Their symptoms encompassed chronic pelvic pain, heavy menstrual bleeding, and increased abdominal volume. After the treatment, patients were clinically reevaluated at 6 months and 12 months when control MRI was performed and the UFS-QoL questionnaire for severe symptoms and quality of life was administered. Of the 63 patients, 9 did not complete follow-up and 2 progressed to a myomectomy by surgical hysteroscopy. The mean uterine volume was 311.02 ± 190.25 cm^3^ (range 98‒900 cm^3^). Of the 111 uterine fibroids, 100 (90.1 %) were intramural, 7 (6.3 %) were subserosal, and 4 were submucosal. The mean size of the fibroids was 5.42 ± 2 cm^3^ (1‒9 cm^3^) (Table 1).

**Table 1:** Demographic and clinical data at study entry.

Characteristics	Mean ± SD (range)
Total number of patients	52
Age (years)	38.52 ± 4.96
Total number of myomas	111
SS	7
SM	4
IM	100
Uterine volume (cm^3^)	311.02 ± 190.25
SSS	50.59
UFS-QoL	42.72

**Fig. 1:**
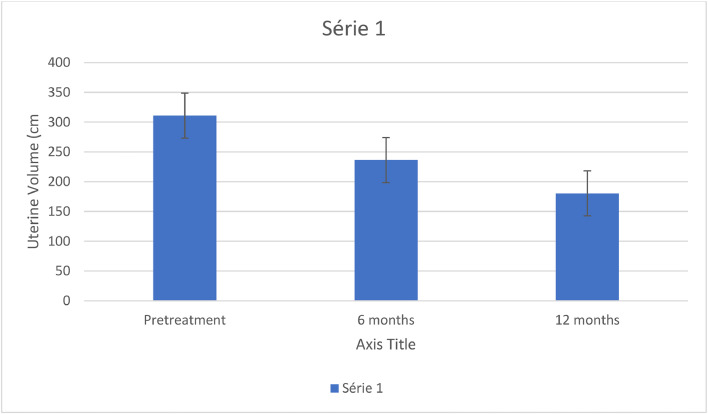
Reduction in uterine volume after MRgFUS.

**Fig. 2:**
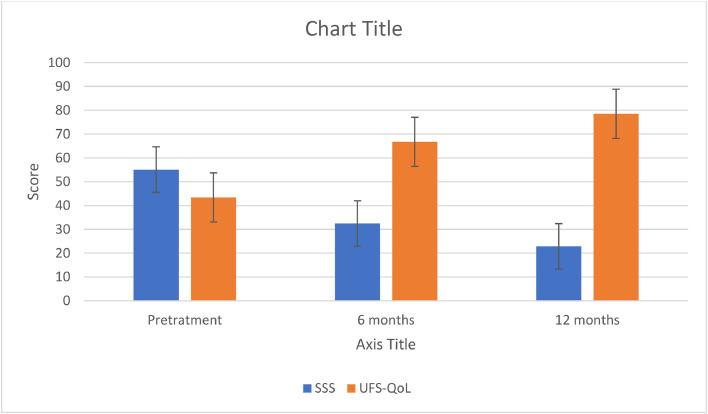
Scores of symptoms and quality of life after MRgFUS.

Fig. 1 shows a significant reduction in the uterine volume after the MRgFUS treatment. The mean volume decreased by 24 % after 6 months of follow-up and by 42 % after 12 months of follow-up. Fig. 2 shows the reduction in symptoms and improvement in quality of life after 6 months and 12 months of follow-up. The mean SSS score fell 43 % after 6 months and 59 % after 12 months. The mean UFS-QoL score rose 54 % in 6 months and 81 % in 12 months of follow-up (Table 2).

**Table 2:** Scores of symptoms and quality of life after MRgFUS.

Score (mean)	Pretreatment	6-months	12-months	p-value
SSS	55.07	32.46	22.86	*p* < 0.0001
UFS-QoL	43.37	66.71	78.55	*p* < 0.0001

The complications were evaluated based on the standards set by the Society of Interventional Radiology Standards of Practice and by the Committee for Classification of Complications by Outcome.^8^ There were no serious complications. Four patients had first-degree burns. No infection cases were reported. Two patients progressed to a myomectomy by surgical hysteroscopy, and one of them underwent total hysterectomy 11 months after the MRgFUS treatment.

## Discussion

Myomectomy is the treatment of choice of some researchers.^1^, ^2^, ^3^ However, it entails a few things that can be avoided, namely recovery time, prolonged hospital stays, high intraoperative complication rates, and possible hysterectomies. In this case series, the authors evaluated the Magnetic Resonance-guided Focused Ultrasound (MRgFUS), which was found to be an effective, promising, and safe technique for the treatment of uterine tumors.^1^, ^2^, ^3^ Since its introduction and approval by the FDA as a therapeutic modality (2004), several studies have been conducted, which have reported improvement in symptoms of leiomyoma patients,^6^ as was the case in this study. Recently, Chen et al. (2018) carried out a large nonrandomized study, in which patients with symptomatic uterine fibroids were treated with MRgFUS.^8^ Of the 2441 recruited women, 1353 underwent the treatment. The statistical analysis showed that the SSS and the UFS-QoL scores improved more rapidly after the treatment.

The variety of myoma types and the signal intensity in the T2 images soon after the treatment may have a bearing on outcomes and symptom improvement.^9^, ^10^, ^11^, ^12^, ^13^, ^14^ In the analysis of volume, the tumors showing a hyposignal in T2 images in MRI tended to have higher necrosis rates and, consequently, greater reduction in leiomyoma volume. This is why the Non-perfused Volume (NPV) of the tumors may be an important predictor of symptom improvement. The reason for this connection is still unknown, but it appears not to depend entirely on tumor volume. Other factors, such as vascularization of the leiomyoma, intramural component, lesion density, and the hormone levels expressed by the tumors may be involved.^12^^,^^15^ It should be emphasized that a limitation of this case series was the lack of access to the NPV rates after the MRgFUS treatment. Nevertheless, the volume reduction of the uteri and the myomas was significant.

Quality of life and symptoms were evaluated with the questionnaire on Uterine Symptoms and Quality of Llife (UFS-QoL). A low score on the SSS, which is part of the UFS-QoL, means improvement in symptoms, whereas a low total score on the UFS-QoL questionnaire points to an enhancement of the patient's quality of life. The results of the present study show that MRgFUS use not only mitigates severe symptoms but also upgrades the quality of life, notwithstanding the types of uterine fibroids and the patients’ ages in this study.^7^

The SSS primarily evaluates symptoms such as those of menorrhagia, and it yields more reliable values, therefore, in cases of patients with intramural or submucosal fibroids. Improvement in quality of life and in bleeding and dysmenorrhea symptoms is also related to a higher rate of tumor necrosis and, therefore, to a greater reduction in the size of the uterus and uterine fibroids. The link between NPV and mitigation of symptoms may also be explained by the association between hypersignal on T2 images of the MRI and greater NPV as mentioned above.^7^^,^^8^^,^^16^

The patients’ follow-up time after MRgFUS was at least 6 months and at most 12 months. This study is limited by the fact that it is a case series with a small number of patients. Nonetheless, the patients showed a reduction in symptoms and in leiomyoma volume, despite the diversity of tumor types. In 12 months of follow-up, the mean uterine volume shrank 42 %. The SSS score, a measure of symptoms, diminished 59 %, and the quality-of-life score on the UFS-QoL questionnaire increased over 80 %.

## Conclusion

This case series suggests that therapy with MRgFUS is viable and effective in mitigating symptoms and improving the patients’ quality of life. A multicenter randomized study comparing MRgFUS with the conventional treatments for uterine myomas, such as myomectomy, would be very useful in providing further scientific evidence of the results of this treatment.

## Ethics approval

Previously authorized by an appropriate ethics committee, and the informed consent was obtained from all individual participants included in the study.

## Funding

The authors did not receive support from any organization for the submitted work.

## Declaration of competing interest

The authors declare no competing interests and no relevant financial or non-financial interests to disclose. On behalf of all authors, the corresponding author states that there is no conflict of interest. The publication of data from this study was also authorized by the participants and the specialized institutional ethics committee.
